# Mucinous adenocarcinoma transformed from tailgut cyst: a case report and review of the literature

**DOI:** 10.3389/fonc.2025.1729627

**Published:** 2026-01-05

**Authors:** Qianyu Guo, Joseph B. Parker, Adam Henderson, Andras Khoor, Michael M. Mohseni

**Affiliations:** 1 Department of Radiation Oncology, Mayo Clinic Florida, Jacksonville, FL, United States; 2 Mayo Clinic Comprehensive Cancer Center, Jacksonville, FL, United States; 3 Feinberg School of Medicine, Northwestern University, Chicago, IL, United States; 4 Robert Lurie Comprehensive Cancer Center, Chicago, IL, United States; 5 Department of Internal Medicine, Mayo Clinic Florida, Jacksonville, FL, United States; 6 Mayo Clinic Alix School of Medicine, Jacksonville, FL, United States; 7 Department of Pathology, Mayo Clinic Florida, Jacksonville, FL, United States; 8 Department of Emergency Medicine, Mayo Clinic Florida, Jacksonville, FL, United States

**Keywords:** tailgut cyst, tailgut cyst adenocarcinoma, tailgut cyst: cystic hamartoma, hindgut, malignant transformation, mucinous adenocarcinoma, retrorectal cyst

## Abstract

Tailgut cysts often present asymptomatically or with nonspecific symptoms. While commonly benign, they may in rare cases be malignant, often transforming into adenocarcinoma. We present a 68-year-old female who presented with a sacral mass and abscess. Upon Emergency Department presentation, her inflammatory markers were elevated as well as her lactate. Initially, the patient was treated for possible infectious etiology with parenteral antibiotics. However, an MRI was obtained that showed concerns for myxoid neoplasm or chordoma. A biopsy was subsequently performed that revealed mucinous adenocarcinoma. Additionally, on imaging there was an enhancing pelvic lymph node concerning for metastatic spread. Tailgut cysts with malignant transformation to adenocarcinoma are rare and are typically treated surgically to obtain clear margins. However, neoadjuvant chemotherapy may be used in patients who have metastatic disease, which was pursued in the course of our patient’s care. This case presentation emphasizes the importance of a wide differential for presacral masses with atypical presentations raising concerns for underlying malignancy. Prompt recognition and intervention is imperative in cases of malignant transformation of tailgut cysts.

## Introduction

Tailgut cysts, also known as retrorectal cystic hamartomas, are rare congenital lesions arising from remnants of the embryonic hindgut. Embryologically, the cloacal membrane covers the distal aspect of the future hindgut at the fourth week of gestation and normally regresses by the eighth week; persistence of this structure leads to a tailgut cyst (1). Histologically, they are composed of various epithelial linings but are distinguishable from teratomas due to the absence of skin or mesenchymal tissue (2). They exist in the presacral space, just posterior to the rectum, and are thus often asymptomatic, but may present with nonspecific symptoms such as perineal pain, defecatory dysfunction, or may be identified during imaging for unrelated concerns (3).

While typically benign, tailgut cysts carry a small risk of malignant transformation, most commonly into neuroendocrine tumor or adenocarcinoma (3, 4). Malignant transformation is often an incidental finding, complicating diagnosis and treatment. In rare circumstances, malignancy may be detected due to a secondary infection, which may manifest as a perianal or pilonidal abscess (5). We report a case of a 68-year-old woman who presented with a spontaneously ruptured sacral mass and presumed abscess, ultimately diagnosed as mucinous adenocarcinoma arising from a tailgut cyst.

## Case

**Figure 1:**
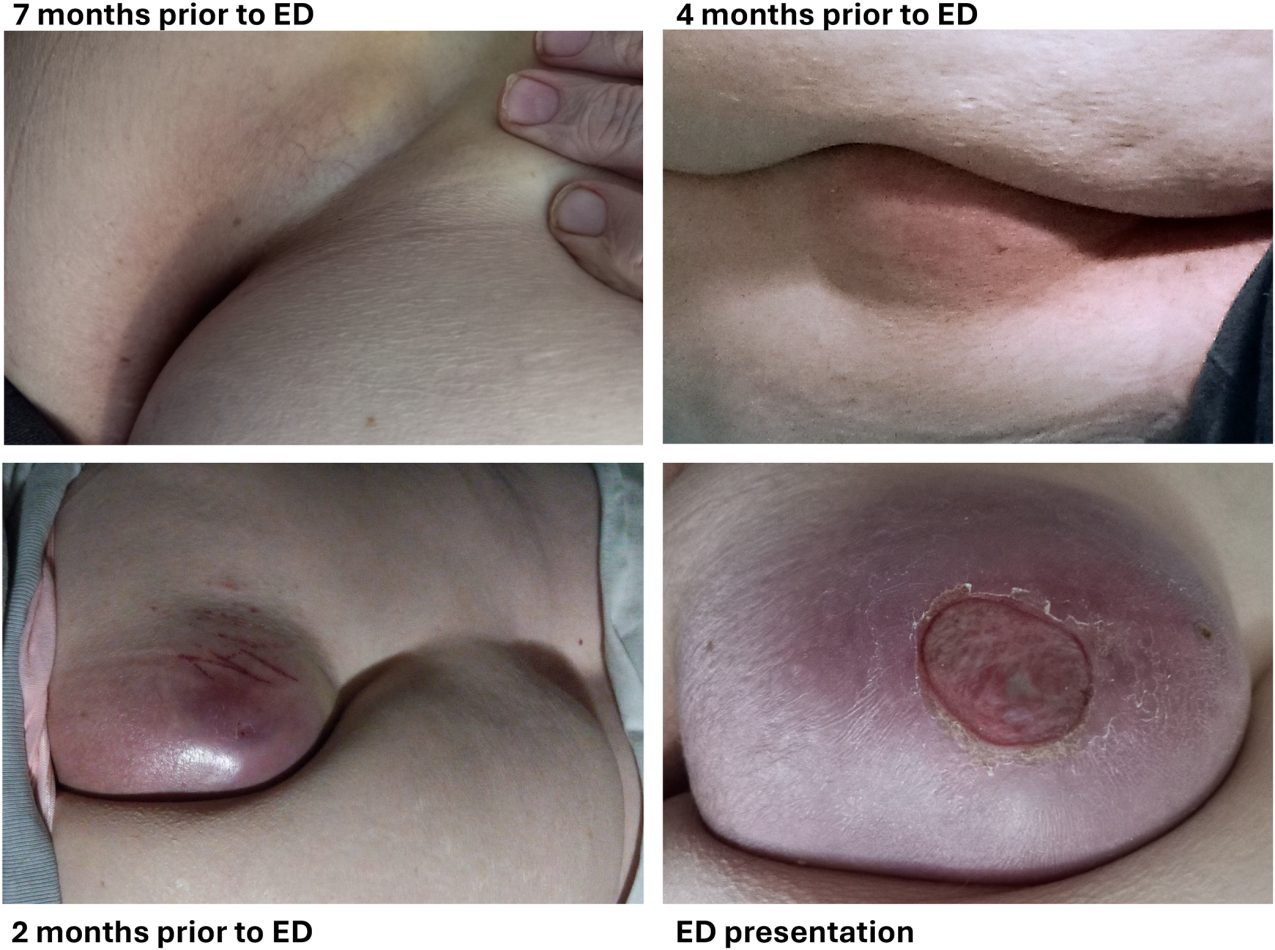
Clinical images. Images provided by the patient which demonstrate the gradual evolution of the lesion over a 7-month period, ultimately progressing to ulceration prior to the diagnosis of mucinous adenocarcinoma. ED, Emergency Department.

A 68-year-old Caucasian female with a past medical history of remote pulmonary embolism, type 2 diabetes, iron-deficiency anemia, and hypertension presented to our institution’s Emergency Department (ED) after experiencing spontaneous rupture of a presumed sacral abscess. The lesion had been gradually increasing in size for 7 months prior to the ED presentation (**Figure 1**). The patient had previously undergone a biopsy at an outside hospital, which revealed acellular mucinous tissue. She was treated with a course of oral amoxicillin/clavulanate but did not experience significant improvement. She denied systemic symptoms such as fever, chills, loss of appetite, or fatigue.

On admission, laboratory results revealed lactic acidosis (lactate 4.2 mmol/L) and elevated inflammatory markers—ESR 62 mm/hr and CRP 11.7 mg/L—consistent with an acute inflammatory or infectious process. The patient had mild anemia (hemoglobin 10.4 g/dL, hematocrit 34.9%) with normal platelet and white blood cell counts. Tumor markers were within normal limits (CA 19-9: 14 U/mL; CA 125: 8 U/mL; CEA: 3.4 ng/mL, slightly above non-smoker reference). CRP levels peaked at 71.5 mg/L on day 4, then declined steadily to 30.5 mg/L (day 7), 21.1 mg/L (day 9), and 23.8 mg/L (day 10), indicating a robust inflammatory response followed by gradual clinical improvement. Blood cultures eventually grew methicillin-sensitive *Staphylococcus aureus* (MSSA), confirming bacteremia. Overall, findings were initially consistent with systemic inflammation due to MSSA bacteremia, mild anemia, and an appropriate biochemical response to treatment without obvious evidence of malignancy.

**Figure 2:**
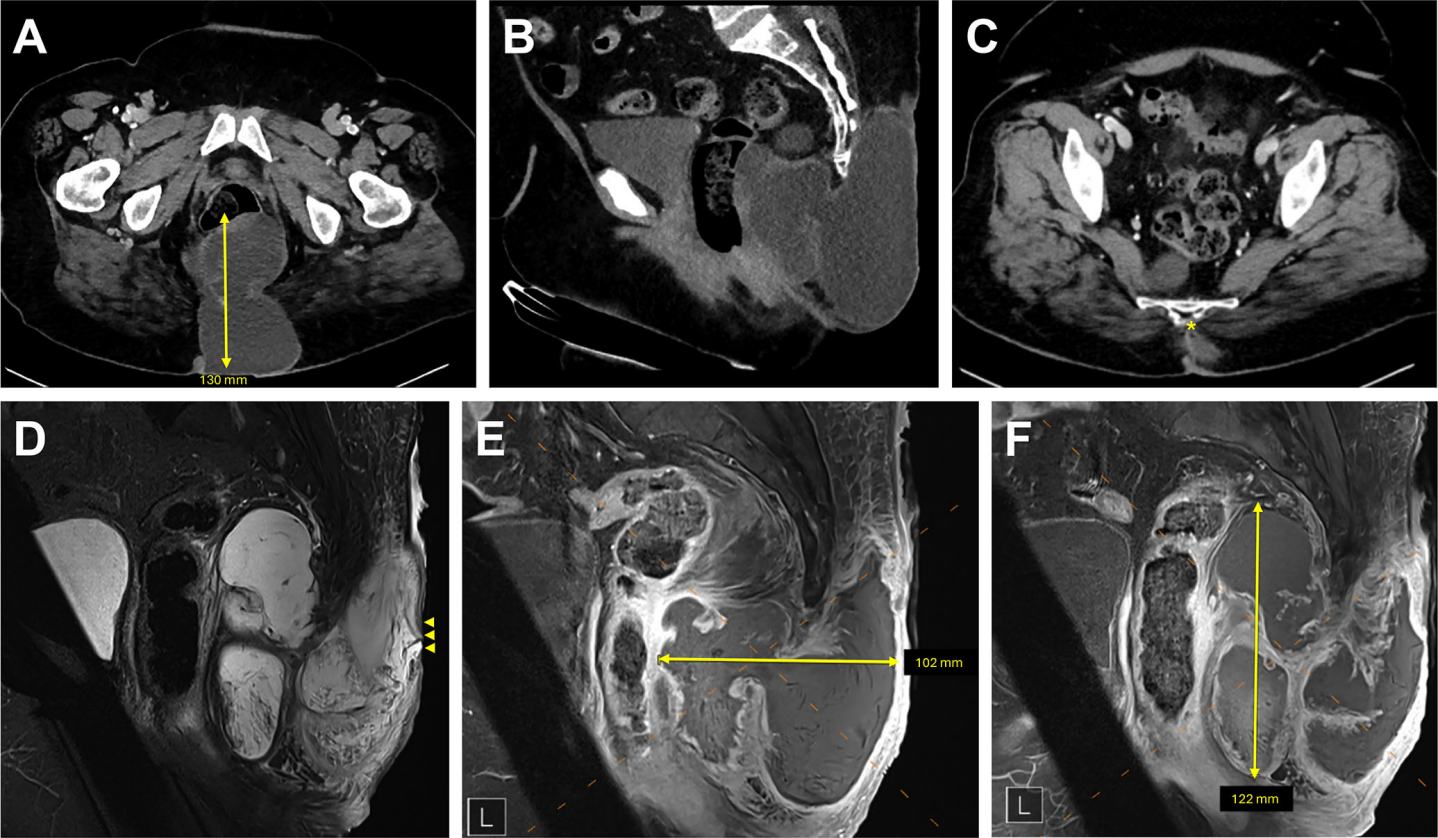
Initial abdominal and pelvic computed tomography (CT) and magnetic resonance imaging (MRI) at diagnosis. **(A)**. Axial CT image showing the mucinous adenocarcinoma measuring 130 mm in maximal diameter (yellow arrow). **(B)**. Sagittal CT image demonstrating extent of tumor in posterior soft tissues. **(C)**. Axial CT image demonstrating possible erosion of the coccygeal tip (yellow star). **(D)**. Sagittal MRI image revealing tumor-associated ulceration (yellow arrowheads). **(E)**. Sagittal MRI image depicting tumor dimension width of 102 mm (yellow arrow). **(F)**. Sagittal MRI image depicting tumor dimensions length of 122 mm (yellow arrow).

A computed tomography (CT) scan of the abdomen and pelvis revealed a multiloculated complex lesion centered at the sacral coccyx with possible bony erosion (**Figures 2A–C**). She was started on empiric intravenous (IV) cefepime and vancomycin. Subsequently, a superficial fluid swab also grew MSSA, prompting a switch to IV cefazolin (Ancef).

Further evaluation with magnetic resonance imaging (MRI) revealed a large presacral and post-sacral mass with features concerning for a myxoid neoplasm or chordoma (**Figures 2D–F**). Additionally, a 1 cm enhancing lymph node on the left pelvic wall raised concern for metastasis, though the malignancy did not appear to originate from the rectum. To obtain a definitive diagnosis, the interventional radiology team performed a CT-guided fine needle biopsy, which identified mucinous adenocarcinoma arising from a tailgut cyst on histology.

**Figure 3:**
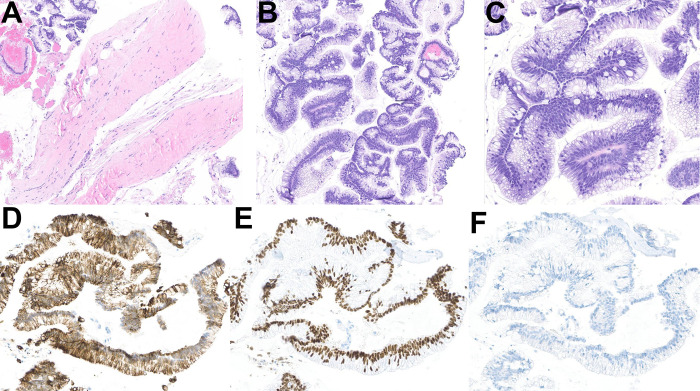
Histopathological and immunohistochemical findings of mucinous adenocarcinoma arising in a tailgut cyst. **(A)** Hematoxylin and eosin-stained section of the biopsy showing a portion of a cyst wall with detached fragments of mucinous adenocarcinoma in the left upper and right lower corners (original magnification x79). **(B, C)** Hematoxylin and eosin-stained sections showing details of the mucinous adenocarcinoma at higher magnification (original magnifications x100 and x200). Immunohistochemical studies revealing that the tumor cells are positive for CK20 **(D)** and CDX2 **(E)** and negative for CK7 **(F)**, consistent with the diagnosis of mucinous adenocarcinoma (original magnifications x200).

The histopathological and immunohistochemical findings are shown in **Figure 3**. Histologic sections showed a portion of a cyst wall and multiple fragments of atypical mucinous epithelium with mucin-rich glands (**Figures 3A–C**) supporting the diagnostic features of an adenocarcinoma. In immunohistochemical studies, the neoplastic cells were positive for CK20 and CDX2 and negative for CK7, supporting an intestinal immunophenotype (**Figures 3D–F**). Taken together, the clinical, radiological, and histopathological findings were consistent with mucinous adenocarcinoma arising in a tailgut cyst.

Given the initial MSSA-positive culture from the mucinous tissue, the infectious disease team recommended a total 10-day course of IV cefazolin. Repeat biopsy cultures showed no microbial growth. During hospitalization, the patient also received IV iron (Venofer) for iron-deficiency anemia and fluconazole (Diflucan) for vulvovaginal candidiasis.

The patient completed concurrent chemoradiation 3 months after the initial ED visit, receiving external-beam radiotherapy to a total dose of 50 Gy in 25 fractions, along with capecitabine 1,000 mg twice daily by mouth during radiotherapy. The colorectal surgery team advised that complete tumor resection would require a proctectomy with permanent colostomy. The patient declined surgical intervention (due to a history of childhood psychological and sexual trauma), acknowledging that this choice would limit the likelihood of achieving the optimal oncologic outcome. Follow-up CT at an outside facility 3 months after chemoradiation demonstrated suboptimal response with ongoing mass measuring 9.2 x 4.0 x 7.2 cm.

## Discussion

Tailgut cysts were first described in 1885 (5), as an entity presenting in the retro-rectal space (also referred to as the presacral space), being posterior to the rectum and anterior to the sacrum and coccyx. They are embryological remnants of the postnatal portion of the hindgut, and failure of regression of this structure leads to cyst formation (6). The incidence of tailgut cysts can be difficult to determine, though data from a review at the Mayo Clinic from 1960–1979 suggested an incidence of 1:40,000 with a strong female predominance (7). Other large reports, including a case series of 53 cases (8) and a systematic review of 196 cases (4), confirm between three- and four-to-one female-to-male predominance with the average presentation in midlife, and approximately half of these patients were asymptomatic (8). Due to a risk of malignant transformation, diagnosed tailgut cysts are generally surgically excised. The malignancy rate quoted has been variable, with a recent Mayo Clinic review of 73 patients suggesting an 8% rate of cancer in these patients (9). The case report reviews likely overestimate the true prevalence, as the authors themselves acknowledge, given the tendency for case reports to emphasize rare or striking presentations (4, 8). In contrast, the data from the Mayo Clinic indicate a much lower malignancy rate of approximately 8%, while other studies attempting to characterize the rate of malignant transformation have reported a broader range, from 1.9% to 26.6% (9).

The common neoplasms are adenocarcinoma, neuroendocrine tumors and carcinoid tumors, but other potential transformations include transitional cell carcinoma or squamous cell carcinoma (2–4, 10). Of note, Prasad reported 2 out of 5 tailgut cyst cases that transformed into malignancy, with one becoming mucinous adenocarcinoma and one become neuroendocrine tumor (2). Given the deep location and nonspecific presentation of mucinous adenocarcinoma, lesions may go undiagnosed until they reach an advanced stage (2).

We reviewed 23 additional mucinous adenocarcinoma cases in the literature, summarizing their initial clinical presentations, tumor marker profiles, immunohistochemistry findings, treatment approaches, and clinical outcomes (**Table 1**). Most patients presented with non-specific symptoms such as lower abdominal, pelvic, or rectal discomfort, changes in bowel habits (including subacute onset of constipation), or persistent purulent drainage that was initially suspected to represent an abscess and treated empirically with intravenous antibiotics. Treatment strategies varied, with patients receiving chemotherapy, radiotherapy, surgery, or a combination of multiple modalities. Reported follow-up durations ranged from 7 months to 4 years, with no documented disease recurrences, supporting the relatively indolent clinical course of mucinous adenocarcinoma in these cases.

**Table 1:** Literature review summary for cases of mucinous adenocarcinoma.

Age/gender	Anatomic site	Key presentation features	Tumor markers	Histology	Treatment	Disease-free survival
67yr/M (11)	Left anterior perineum	Abscess 10yr ago → Abscess 3yr ago → Diagnosis	N/A	Well-differentiated adenocarcinoma, abundant mucin	WLE→ Adjuvant capecitabine plus EBRT 50.4Gy	28 months
57yr/F (10)	Anal canal	Mucoid fluid discharge 5yr ago → Removal of 3 jelly-like masses 4yr ago → Increasing anal discomfort 3yr ago & diagnosis of mucinous adenocarcinoma	↑CEA=30 ng/mL	Mucinous adenocarcinoma	Neo-adjuvant concurrent capecitabine plus EBRT 50Gy in 25 fractions → APR (ypT3ypN0)	24 months, no progressive disease
62yr/M (17)	Left buttock	Recurrent abscesses of buttock for 3 years → Multiple incision & drainage → Formation of scars and nodules → Serous discharge	CEA=4.93ng/ml, ↑CA19-9 = 39.69U/ml	Mucinous adenocarcinoma, CK7-, CK20+, CDKX2+	Drainage & WLE (patient refused APR and CRT)	24 months, no disease recurrence
37yr/F (23)	Perineal	Remote history of recurrent perineal abscess & drainage → Perineal lesion → Excision (dermoid cyst) → 1yr later, recurrence → Re-excision (mucinous adenocarcinoma)	↑CEA= 37.9ng/ml	Tailgut cyst mixed with mucinous adenocarcinoma	AT-ISR & radiation	N/A
55yr/F (24)	Right ischiorectal fossa	6 months lower back pain → Diagnosis	CEA WNL	Mucinous adenocarcinoma, CK20+, CDX2+, CK7+, GATA3-, ER-, PR-, calretinin-	WLE & radiation	12 months, no recurrence
35yr/F (20)	Suprapubic mass	Months of abdominal discomfort → Diagnosis	↑CEA= 132.69ng/ml	CK7+, CK20+, CDX2+, STATB2+	Laparoscopic resection & Adjuvant 6 cycles of capecitabine + oxaliplatin	18 months, no recurrence
50yr/F (22)	Anal mass	Irregular defecation with small amount of liquid stool for 2+ months → Diagnosis	↑CEA= 79.89ng/ml, ↑CA19-9 = 57.60U/ml	Tailgut cyst mixed with moderately differentiated adenocarcinoma, CK7+, CK20+, CDX2+, Ki67+	Trans- sacrococcygeal resection → Adjuvant radiotherapy	6 months, no recurrence
73yr/F (16)	Right buttock fistula	Right anal abscess and fistula with purulent discharge for decades → 1L foul smelling pus drainage + surgical resection of abscess & fistula → Mucinous discharge → Complex cyst superior and anterior to the coccyx → Radical excision	N/A	SMA+, CK7+, CK20+, Ki67+ >50%	Radical resection	N/A
54yr/F (27)	Retrorectal space	Pelvic & perineal pain of weeks → Diagnosis	N/A	Tailgut cyst mixed with adenocarcinoma, CAM5.2+, CDX2+, CK20+, CK7+	En bloc resection with a posterior approach (Kraske procedure) → Adjuvant CRT (54Gy/30 fractions)	11 months, no recurrence
43yr/F (18)	Retrorectal/presacral mass	Imminent threatened abortion → Presacral mass → Diagnosis	CEA+, OV-125 & OV-632 -	N/A	Surgical resection → Local recurrence 4 years later → Palliative CHT (5-FU + Leucovorin)	Recurrence after 4 years, patient passed from unrelated cause
40yr/F (25)	Presacral mass	Severe perineal pain of 1 month → Diagnosis	↑CEA=159 ng/mL ↑CA19-9 = 2270 U/mL	N/A	En bloc resection with Hartmann’s procedure + coccygectomy/partial sacrectomy (S4) → adjuvant RT → nodal recurrence → 5-FU chemotherapy	Recurrence after 5 months
53yr/F (15)	Retrorectal/presacral mass	Painful defecation & lower abdominal pain → Diagnosis	N/A	N/A	Surgery	N/A
44yr/F (19)	Presacral mass	Pelvic and perineal pain 6 months → Diagnosis	CEA+	Tailgut cyst mixed with adenocarcinoma	Surgery → TNF + raltitrexed infusion into the cysts → 3 cycles of oxaliplatin	N/A
49yr/F (26)	Retrorectal mass	Pelvic and perineal pain 6 months → Diagnosis	N/A	N/A	Surgery → Adjuvant CRT (MacDonald protocol: 5-FU + folinic acid with 45Gy RT for 12 weeks)	4yr follow up, no recurrence
47yr/F (28)	Presacral mass	3 months enlarging presacral mass → Surgery	N/A	Tailgut cyst mixed with adenocarcinoma	N/A	N/A
64yr/F (14)	Retrorectal space	2 months constipation, pelvic pressure, increased urinary frequency → Diagnosis	N/A	Ki67+, p53 overexpression & mutation	Surgery	10m follow up, no recurrence
68yr/F (14)	Retrorectal space	Chronic rectal “fullness” → Diagnosis	N/A	Ki67+, p53 overexpression & mutation	Surgery	29m follow up, no recurrence
40yr/F (21)	Lesion between sacrum and	8 months of urinary frequency and constipation → Diagnosis	N/A	N/A	Lower abdominal → 4 months recovery → RT	No recurrence at publication; follow-up duration unclear
36yr/F (2)	Retrorectal mass	Asymptomatic; retrorectal mass on digital exam during routine physical → Diagnosis	N/A	Tailgut cyst mixed with adenocarcinoma	Surgical excision	24m follow up, no recurrence
56yr/F	Presacral mass (Chhabra et al, 2013, original citation unavailable)	Incidental imaging finding without resection (reason unclear) → Hematuria 3 years later → Diagnosis	CEA- (within normal range)	N/A	Surgery	1.5yr follow up, no recurrence
38yr/F	(Ballantyne, 1932, original citation unavailable)	Discomfort while sitting → Diagnosis	N/A	N/A	Surgical resection	7m follow up, recurrence with metastasis to groin lymph nodes and lung
62yr/F	(Marco et al, 1985, original citation unavailable)	Discomfort upon sitting with a mass since childhood → Diagnosis	N/A	N/A	Surgical resection	20m follow up, no recurrence
63yr/F	(Levert et al, 1996, original citation unavailable)	Discomfort upon sitting with 9 cm mass on CT scan à Surgery	N/A	N/A	Surgical excision	5-year follow up, no recurrence

In our case, the initial presentation mimicked an infected sacral abscess, delaying recognition of the underlying malignancy. The presence of acellular mucin in the initial biopsy was an early clue, but definitive diagnosis required targeted imaging and tissue sampling. A similar patient was reported in the literature, where the patient presented with perineal abscesses. In this case, the patient had a presumed abscess occurring 10 years prior to presentation resolving after incision and drainage, and another abscess occurring 3 years prior to presentation that did not respond to incision and drainage eventually requiring draining seton placement. This patient eventually presented with palpable left anterior perineal mass with biopsy confirming mucinous adenocarcinoma (11). They received adjuvant capecitabine and external beam radiation therapy (50.4 Gy) postoperatively, and remained free of disease as of 28 months (11).

The management of mucinous adenocarcinoma arising from a tailgut cyst typically involves a combination of surgery, chemotherapy, and radiation therapy. Complete surgical resection with clear margins is the preferred treatment when feasible. However, in cases with metastatic potential or extensive local invasion, neoadjuvant chemoradiation may be necessary before considering resection (12, 13). Among 20 previously reported cases, six were treated with surgery alone (2, 14–17), three with surgery and chemotherapy (18–20), four with surgery and radiotherapy (21–24), five with surgery and chemoradiotherapy (10, 11, 25–27), and one case did not specify the treatment approach (28). In our patient, the presence of an enhancing pelvic lymph node raised concern for metastatic spread, supporting the decision for neoadjuvant therapy before surgery.

The infectious component of this case also posed a diagnostic challenge. The initial MSSA-positive culture from the mucinous tissue raised the question of superimposed infection versus contamination. However, repeat cultures from a deeper biopsy site showed no bacterial growth, suggesting that the malignancy itself may have been the primary driver of inflammation rather than an active infection. This highlights the importance of correlating microbiologic findings with imaging and histopathologic results to avoid unnecessary prolonged antibiotic therapy.

Our patient’s case underscores the diagnostic complexity of presacral masses and the need for a high index of suspicion for malignancy in patients with atypical presentations. Early recognition and multidisciplinary management are essential to optimizing outcomes for these rare but aggressive tumors.

Furthermore, this case highlights the complex psychosocial challenges that can arise when surgical management involves a permanent colostomy. For some patients, the prospect of living with a colostomy bag can cause significant emotional distress, affect body image, and trigger feelings of loss of autonomy. In this case, the patient’s history of trauma added further complexity to treatment planning, influencing her decision-making process and tolerance for certain interventions. Our social work team, together with the oncology, surgical, and radiation teams, provided coordinated multidisciplinary support to address the patient’s psychological needs, ensure informed decision-making, and respect her autonomy while optimizing her overall care.

## Data Availability

The original contributions presented in the study are included in the article/supplementary material. Further inquiries can be directed to the corresponding author.
